# Reversal of isolated unilateral optic nerve edema with concomitant visual impairment following blunt trauma: a case report

**DOI:** 10.1186/1752-1947-2-50

**Published:** 2008-02-18

**Authors:** Marc Maegele

**Affiliations:** 1 Department of Trauma and Orthopedic Surgery, Intensive Care Unit (ICU), University of Witten/Herdecke, Cologne-Merheim Medical Center (CMMC), Ostmerheimerstr. 200, D-51109 Cologne, Germany

## Abstract

**Introduction:**

Serious injury to the optic nerve is an uncommon entity but may result in permanent visual disability. Isolated trauma of the optic nerve is usually associated with blunt skull trauma involving fractures of both skull and optic canal, but may also occur from blunt ocular trauma.

**Case presentation:**

We report a woman who developed isolated unilateral optic nerve edema with corresponding visual deficits after a rear-end collision accident. She was treated with corticosteroids and had a favourable outcome.

**Conclusion:**

The approach described here was successful in this case but the current body of evidence still lacks a validated approach to the management of traumatic optic neuropathy and each case needs to be individually assessed.

## Introduction

Serious injury to the optic nerve is an uncommon entity but may result in permanent visual disability [1]. International rates vary according to the country with rates depending on the occurrence of causative events, for example non-fatal motor vehicle accidents and aggravated assaults. In the United States, traumatic optic neuropathy occurs in 0.5–5% of patients with closed head injuries and in 2.5% of those with midfacial fractures [2]. Data from Germany indicates impairment or loss of vision due to optic nerve injury occurs in approximately 10% of patients with craniofacial fractures [3]. Kallela et al.[4] analyzed clinical and computerized tomography findings from 10 patients with post-traumatic optic neuropathy after maxillofacial blunt trauma. In their review the number of blind eyes was 14 and all patients suffered from midfacial fractures. Isolated trauma of the optic nerve is usually associated with blunt skull trauma involving fractures of both skull and optical canal, but may also occur from blunt ocular trauma [5]. We report on a woman who developed isolated unilateral optic nerve edema with corresponding visual deficits after a rear-end collision accident. She was treated with corticosteroids and had a favourable outcome.

## Case presentation

**Figure 1:**
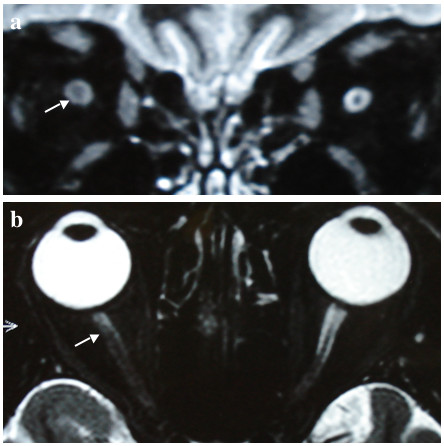
Magnetic resonance tomography shows isolated unilateral distension of the right optic nerve (arrows in panels a and b).

A 45-year-old female was admitted to the emergency department (ED) following a rear-end collision accident. At the scene the patient was awake but somewhat somnolent. Her circulatory function was compensated with a blood pressure (BP) of 150/80 mmHg and she complained of back pain. Following initial assessment the patient was transferred via helicopter to our level 1 trauma centre for further evaluation and treatment. Upon arrival in our trauma bay the clinical picture was unchanged. Detailed clinical assessment including laboratory tests, ultrasound, radiology and computed tomography (CT) was negative and the patient was transferred to our intensive care unit (ICU) for observation. Within one day her cognitive function had returned to normal and the patient was transferred to one of our normal wards. On day 2 following the trauma the patient complained of blurred vision. Ophthalmology assessment revealed a visual field loss affecting the right lower quadrant on confrontation field testing. Clinical eye examination further revealed a visual acuity for the right eye of 0.5 decimal (LogMAR 0.30, Snellen ratio 20/40) and for the left eye of 0.8 decimal (LogMAR 0.1, Snellen ratio 20/25). Pupil testing indicated an afferent defect of the right eye. There was no history of eye disease prior to the accident. Imaging studies, including magnetic resonance imaging (MRI) of the orbit, showed an isolated unilateral distension of the right optic nerve with edematous soaking of the adjacent retro-orbital fat (Figure 1). There was no fracture of the skull or of the optic canal and no intracranial pathology was noted. High-dose corticosteroids were administered for three consecutive days and then reduced, i.e. prednisone 250 mg IV for three days, reduced to 100 mg IV and stopped. The patient's symptoms responded quickly to this approach. Repeated eye examination after one week showed normal testing results for pupillary function and confrontation fields, and visual acuities returned to 1.0 decimal on both eyes (LogMAR 0.00, Snellen ratio 20/20).

## Discussion

The intra-orbital segment of the optic nerve is usually spared from injury due to its laxity and buffering by the surrounding fat and extraocular muscles. The intracranial segment is protected by the surrounding brain and bone as well as the fact that shearing forces are usually absorbed by the intracanalicular segment thus not reaching the intracranial segment. Some investigational studies have shown that blows to the malar and frontal areas are transmitted mostly to the optic foramen[2]. These forces may cause compression, shearing, contusion and stretching injuries to the optic nerve, even in the absence of a fracture. Furthermore, the sheath of the optic nerve is firmly attached to the optic canal, and the canal itself is a closed space, not flexible to any edema or hemorrhage [2,6].

The mechanisms of trauma frequently associated with traumatic optic neuropathy comprise motor vehicle accidents, as shown in our case, but also bicycle accidents, falls, assaults, penetrations, recreational sports injuries, or surgical intervention in the case of orbitofacial fracture repair.

The pathophysiology of post-traumatic optic neuropathy is poorly understood. Most commonly, traumatic optic neuropathy occurs as an indirect event during or shortly after blunt trauma to the superior or lateral orbital rim, the frontal area, or the cranium. Trauma-associated compression forces are transmitted through the orbital bones to the orbital apex and the optic canal. Contusion of the intracanalicular optic nerve axons and pial microvasculature leads to local optic nerve ischemia and edema. Edematous ischemic axons result in further neural compression, induce a postive feedback loop and may thus trigger the development of an intracanalicular compartment syndrome with further necrosis and infarction[6]. Vascular insufficiency may also contribute to the development of traumatic optic neuropathy. During the initial phase, hemorrhage into the nerve, or into any layer of its sheath, laceration and contusion necrosis may occur as a result of the shearing forces.

Secondary damage may not be present initially but may occur later resulting from compromised blood supply to the optic nerve, e.g. following chronic edema, hemorrhage or angiospasm [5]. Our patient had been involved in a rear-end collision accident with a combination of a significant deceleration momentum obviously inducing a sudden and forceful shear on the optic nerve, and a physical impact to the head which hit against the window of the vehicle. Similar but more dramatic scenarios have been described indicating that refractory evulsion of the optic nerve, with similar morphological features as presented here, but also poorer clinical prognosis leading to blindness may occur [7].

Sudden and forceful rotational movements of the eye can tear off the optic nerve at its globe entry level. Post-traumatic loss of vision may also manifest up to two months after the initial impact leading to delayed diagnosis and unresponsiveness to treatment [8].

Clinical assessment should include testing of visual acuity, extraocular muscle motility and papillary reactivity, visual field assessment and direct/indirect ophthalmoscopy. Detailed gonioscopy will rule out potential confounding anterior segment pathologies. The pertinent findings upon clinical examination are impaired visual function and an afferent pupillary defect on the swinging flashlight test, both with an eye that appears normal [6]. The patient reported here displayed a substantial decrease in visual acuity together with a visual field loss to the right lower quadrant upon confrontation field testing. Visual evoked potentials (VEPs) to flash stimulation and the electroretinogram (ERG) might be supportive in unresponsive patients in the immediate aftermath of the traumatic event [3,9]. Altenmüller et al.[3] reported good correlation of initial VEPs with the visual acuity and visual fields examined after patients had regained consciousness.

The role of neuroimaging remains controversial and practice varies between institutions. While some colleagues request computed tomography (CT) and/or magnetic resonance imaging (MRI) for diagnosis, others limit these to patients with progressive visual deterioration or if therapeutic interventions are being considered. The clinical value of neuroimaging in traumatic optic neuropathy is further debatable since there is no consistent correlation between the finding of an optic canal fracture, the severity of visual loss and the prognosis for visual recovery.

Recently ultrasonography has been advocated to screen and detect abnormalities in optic nerve diameter in patients who have experienced head trauma that could involve the optic nerve [8,10], including its use in bedside emergency department conditions [11].

Currently, there is no validated approach to the management of traumatic optic neuropathy. The International Optic Nerve Trauma Study [12] was initiated to compare the visual outcomes of patients observed without treatment with those of patients treated with corticosteroids and of patients treated with optic canal decompression surgery. This multicenter, comparative, interventional but non-randomized trial comprised 133 patients with traumatic optic neuropathy from 16 countries. Treatment decisions were according to the investigators' customary practice and no specific protocols for corticosteroid treatment or surgical technique were followed. The results showed that visual acuity improved in32% of patients treated with surgery, in52% of patients treated with corticosteroids, and in 57% of untreated patients. Thus, there was no clear benefit observed for either corticosteroid therapy or optic canal decompression. The results further showed that neither the dosage or timing of corticosteroid treatment nor the timing of optic canal decompression were associated with an increased probability of improved visual acuity. The authors concluded that neither corticosteroid therapy nor optic canal decompression should be considered the standard of care for patients with traumatic optic neuropathy and that therapeutic decisions should be made on an individual patient basis. In the present case, the patient's symptoms quickly responded to corticosteroid therapy but considering the results from the International Optic Nerve Trauma Study, this patient may have improved without any specific therapy whatsoever as well.

The rationale for intravenous corticosteroids for the treatment of traumatic optic neuropathy was derived from the results of the National Acute Spinal Cord Injury Study 2(NASCIS 2). The NASCIS 2 was a multicenter clinical trial that evaluated patients with acute spinal cord injury treated with placebo, methylprednisolone, or naloxone. Pharmacologically, corticosteroids are considered to reduce microvascular spasm and soft tissue edema via stabilization of the microvascular circulation and calcium homeostasis, thereby enhancing bloodflow and reducing cell death. The study showed that methylprednisolone started within 8 hours of injury was associated with a significant improvement in both motor and sensory function compared to patients treated with a placebo. Although widely accepted, the question whether corticosteroids are of similar effect in the treatment of traumatic optic neuropathy is unproven.

The majority of case reports and series with corticosteroids in traumatic optic neuropathy are retrospective, non-consecutive, non-randomized, and uncontrolled. Meanwhile, several non-clinical studies have questioned the therapeutic benefit associated with corticosteroids in acute traumatic optic neuropathy [13,14]. The results from the CRASH-trial indicated an even higher risk of mortality in patients with head injury treated with high-dose corticosteroids. The author acknowledges that if a clinician chooses to administer corticosteroids that have no proven benefit and the patient dies, a medicolegal issue may arise because of the results from the CRASH-trial.

One may speculate that the pure white matter optic nerve is not pharmacologically affected in the same manner as the mixed white and gray matter spinal cord.

Surgical optic nerve decompression has similarly been advocated to improve visual prognosis in traumatic optic neuropathy. Recently, YuWai Man and Griffiths [15] assessed the effects and safety of surgical interventions in the management of traumatic optic neuropathy. Based upon only small and retrospective case series, and the wide range of surgical interventions used, they encountered considerable difficulties in comparing the body of evidence available. Given the relatively high rate of spontaneous visual recovery they concluded that there is no evidence that surgical decompression of the optic nerve provides any additional benefit [15]. However, in selected cases in which orbital bone fragments or foreign bodies impinge on, but do not transect the optic nerve, surgical intervention may be indicated. In any case, one should be aware of the fact that surgical intervention carries a definite risk of complications such as collateral damage to structures of the orbital apex as well as other intracranial structures, or iatrogenic direct and indirect optic nerve damage, the latter via disruption of the pia, as well as postoperative cerebrospinal fluid leaks and meningitis. Similar to corticosteroids, the use of surgery in traumatic optic neuropathy remains controversial and each case needs to be individually assessed.

## Conclusion

The coincidence with the traumatic event, the absence of any eye pathology prior to the traumatic event and the exclusion of any alternative cause for an optic nerve swelling prompted the diagnosis in this patient of a post-traumatic unilateral optic nerve contusion with corresponding visual deficit which quickly responded to steroid therapy. This approach was successful in the case reported here but the current body of evidence still lacks a validated approach to the management of traumatic optic neuropathy and each case needs to be individually assessed. There is a need for a large, prospective, randomized controlled trial to assess the different therapeutic approaches in traumatic optic neuropathy but such a trial may be challenging given the low frequency of the condition and the difficulties inherent in randomizing patients.

## Competing interests

The author(s) declare that they have no competing interests.

## Authors' contributions

MM assembled all relevant data to this case report, performed the literature review and drafted the manuscript.

## Consent

Written informed consent was obtained from the patient for publication of this case report and any accompanying images. A copy of the written consent is available for review by the Editor-in-Chief of this journal.
